# The effect of cane length and step height on muscle strength and body balance of elderly people in a stairway environment

**DOI:** 10.1186/1880-6805-33-36

**Published:** 2014-12-21

**Authors:** Zi Ying Li, Chinmei Chou

**Affiliations:** Department of Industrial Engineering & Management, Yuan Ze University, 135 Yuan-Tung Road, Chung-Li, 32003 Taiwan ROC

## Abstract

**Background:**

It has been reported that 75% of stairway accidents occur while descending stairs. Using a cane can help to prevent older people and those with limited mobility from falling. However, studies have shown that two-thirds of older cane users use a cane that is longer than the recommended length, which may cause unnecessary muscular loads. This study aims to assess balance and muscular load in older people descending different height steps with different cane lengths.

**Methods:**

Nine participants (5 males and 4 females) aged over 65 years participated in this study. Cane length and stair height were independent variables. Electromyography signals were recorded from the biceps brachii of the arm that usually held the cane and from both gastrocnemius muscles. In addition, the center of pressure (CoP) was assessed as an indicator of balance in older people descending a step.

**Results:**

Descending from higher steps resulted in the use of greater arm and leg strength at the time of first foot contact. However, cane length did not affect any of the root mean square values. In addition, the CoP Stabilometric Parameters showed that mean distance, antero-posterior mean distance, total excursions, antero-posterior total excursions, mean velocity, and antero-posterior mean velocity were significantly affected by step height, but not by cane length.

**Conclusions:**

If cane length is within the currently suggested range, then it has little effect on the force load on the arm and legs when descending a step. Step height has a greater effect than cane length on the strategies used by older people to maintain stability.

## Background

As people grow older, they experience functional declines in movement, consciousness, and cognition [1, 2]. Age-related declines in movement result from changes in muscle tissue, including reduction in the quality, quantity, size, and cross-sectional area of the skeletal muscles [3]. Muscle quality and quantity decrease by approximately 30% to 40% from age 50 to 80 years [4, 5]. This phenomenon often leads to difficulty walking, changes in gait, and an increased risk of falling, which is a common cause of death in older people. Many studies have examined the relationship between aging and falls [6–8]. The frequency of falls increases with aging, reaching 30% to 40% after 60 years of age [9]. One study of older subjects in the United Kingdom and the United States showed that falls were the main cause of accidental death in older people [10], and approximately one-third to one-half of older people fall down stairs once a year [11–13]. Additionally, another investigation showed that 75% of stairway accidents occurred while descending stairs [14], with a 3:1 hazard ratio between walking down and walking up stairs.

Using a cane can reduce the risk of falling, while also reducing pressure on the lower limbs [15]. Additionally, using a cane is an ideal means of improving the confidence of older people during walking [16]. The correct use of a cane can offset 20% to 30% of an individual’s body weight [17]. However, although recommended cane lengths result in only 20° to 30° of elbow flexion, it has been reported that two-thirds of older cane users use a cane that is too long [16].

Excess cane length inhibits the transfer of arm strength or body weight to the cane, causing unnecessary pressure on the triceps. It can also increase elbow flexion and lead to an improperly raised shoulder. In contrast, a shorter cane length can cause the user to lean forward while standing or walking. In short, a cane of the wrong length will make the user feel uncomfortable and significantly increase energy expenditure [15, 16]. Research indicates that the proper cane length is either the distance from the ground to the greater trochanter or the distance from the ground to the wrist crease [15, 18, 19].

There is considerable disagreement over the relationship between cane length and usage and fall occurrence [18]. Experiments targeted on different populations, genders, and ages using a variety of cane lengths in different situations have failed to provide definitive results. Jones et al. [20] measured participants’ heart rates to evaluate the physiological effects of different cane lengths (Type 1: from floor to greater trochanter, average: 83.1 cm; Type 2: from floor to distal wrist crease, average: 78.6 cm; Type 3: the individual’s Height(cm) × 0.45 + 0.087(m), average: 79.7 cm); their results showed no effect of cane length on heart rate. Another study targeted stroke patients [21]; the participants were divided into two groups based on whether their arm length was shorter or longer than 50% of their height and both groups used the same length canes (from floor to greater trochanter). The results indicated that there was more pressure on the heels of the feet in the group with shorter arm length [21]. To date, there is no definitive evidence for the correct cane length for older people during walking on a level surface. Moreover, research on walking down stairs with a cane has been limited, despite the high risk of the stair environment. If older people use a cane improperly, it might increase pressure on the body and even lead to falls with potentially serious consequences. Thus, the purpose of this study was to determine the effects of various cane lengths on balance and muscle loading in older people when descending steps of different heights. Specifically, we tested the following two hypotheses, i) in older persons, the use of a cane for descending from a high step may result in smaller muscle loads and better balance than level walking without a cane, and ii) different cane lengths will affect balance and muscular loads when descending a step.

The results will offer older people some guidelines for proper cane length selection under a variety of conditions, and as a result may help them feel more comfortable while walking and descending stairs.

## Methods

### Study design and participants

The participants in this cross-sectional study were recruited between October and December 2013 from the Suang-Lien Elderly Center in New Taipei City, Taiwan. Five males and four females participated in this study. Their mean age was 82.78 ± 7.66 years, height was 160.89 ± 8.82 cm, and weight was 60.50 ± 12.87 kg. Six of the participants routinely used a cane and three of these had osteoarthritis. The other participants occasionally used a cane (Table 1). All participants had functionally normal eyesight, either with or without corrective lenses. Any potential participants who had a disease that affected balance were excluded. Prior to inclusion in the study, prospective participants were assessed using the Berg Balance Scale; only those with scores ≥24 were included in the study. All participants gave their written informed consent and all experimental protocols were approved by the institutional review board of the Research Ethics Office of National Taiwan University.

**Table 1:** **Participant data**

Participant	Gender	Use cane usually	Osteoarthritis	Age	Height (cm)	Weight (kg)	Cane 1 (cm)	Cane 2 (cm)	Cane 3 (cm)
1	Female	Yes	No	87	150	42.5	76	73	70.5
2	Female	Yes	Yes	80	150	45	80	76	70.5
3	Female	No	No	86	148	60	78	73	70.5
4	Female	Yes	Yes	93	168	58	85	74	76
5	Male	Yes	No	85	166	67	86	78	76
6	Male	Yes	No	83	167	68	90	85	76
7	Male	Yes	Yes	65	168	86	90	85	76
8	Male	No	No	81	168	60	88	85	76
9	Male	No	No	85	163	58	83	80	76
Average	Female = 4	Yes = 6	Yes = 3	82.78	160.89	60.50	84.00	78.78	74.17
Standard Deviation	Male = 5	No = 3	No = 6	7.66	8.82	12.87	5.12	5.19	2.75

### Variables

The independent variables in this study were cane length and step height. Three cane lengths were tested as follows: the length of Cane 1 was from the floor to the greater trochanter [15, 18, 19], the length of Cane 2 was from the floor to the distal wrist crease [22], and Cane 3 was a shorter cane that resulted in approximately 20° of elbow flexion when held in a neutral, upright position; the final cane category was No Cane. Step heights were 0 (ground level), 18, 27, and 33 cm. All tests consisted of descending a single step 45 cm deep, 90 cm wide, and of varying height. Dependent variables were the center of pressure (CoP), measured by a low-pass filter force plate, and electromyography (EMG) signals (NeXus-10, 1,024 Hz, MindMedia, Netherlands). EMG patches were attached over the biceps brachii of the arm that usually held the cane and both gastrocnemius muscles.

### Procedure

**Figure 1:**
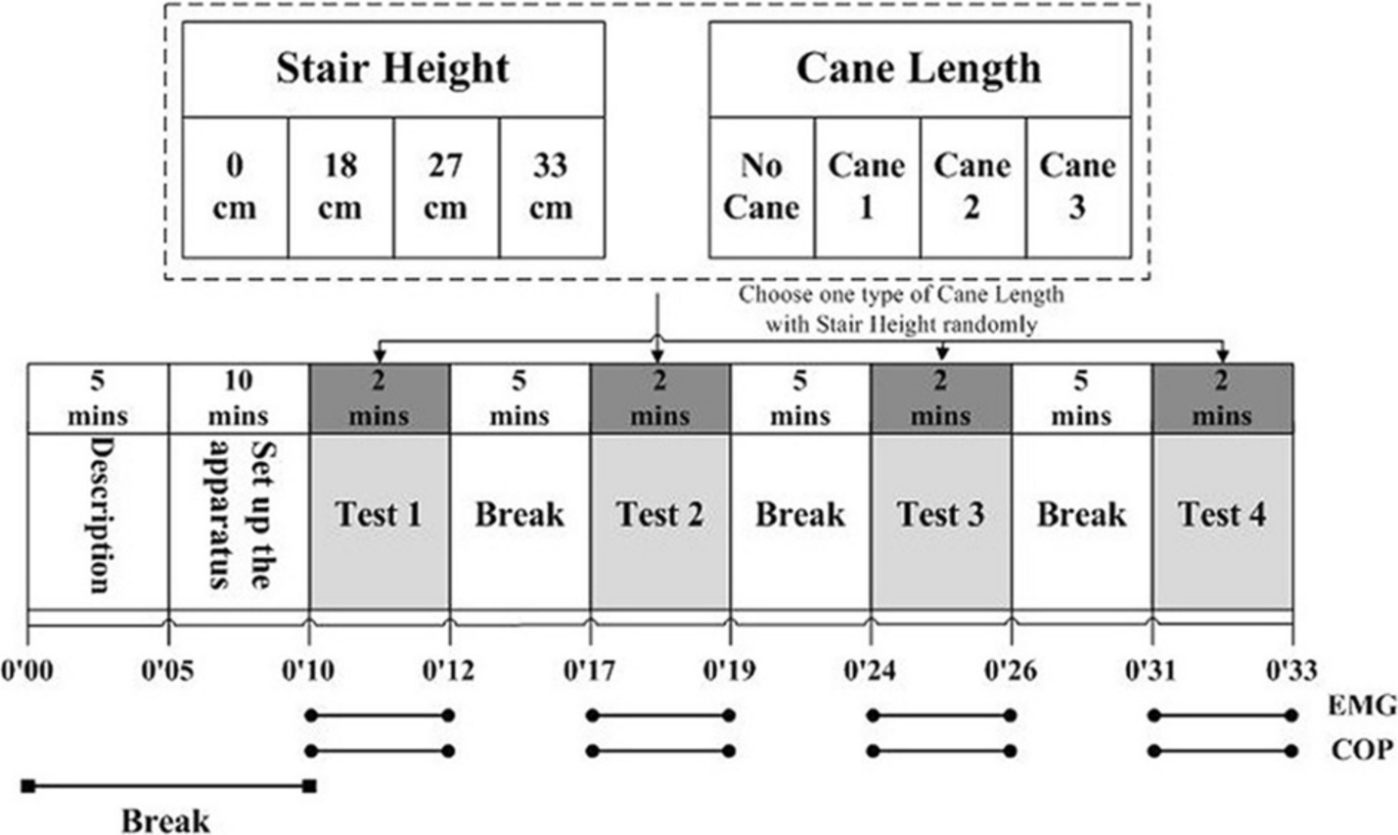
**Experimental procedure.**

The total experiment time was approximately 40 minutes (Figure 1). Participants were asked to sit down and rest for 15 minutes while the apparatus was set up. After the 15 minute rest, the participants completed tests 1 through 4. In each test, participants stood on the step and stepped down onto the force plate when asked to by the researcher. A handrail was available during each test for the participants to use at their discretion. After stepping down, the participants stood on the force plate for at least 10 seconds and then repeated the test. Each test combination of cane length and step height was repeated twice. All participants completed four randomly selected tests of different step heights and cane lengths each day, and finished all 16 combinations within four sessions.

### Data analysis

**Figure 2:**
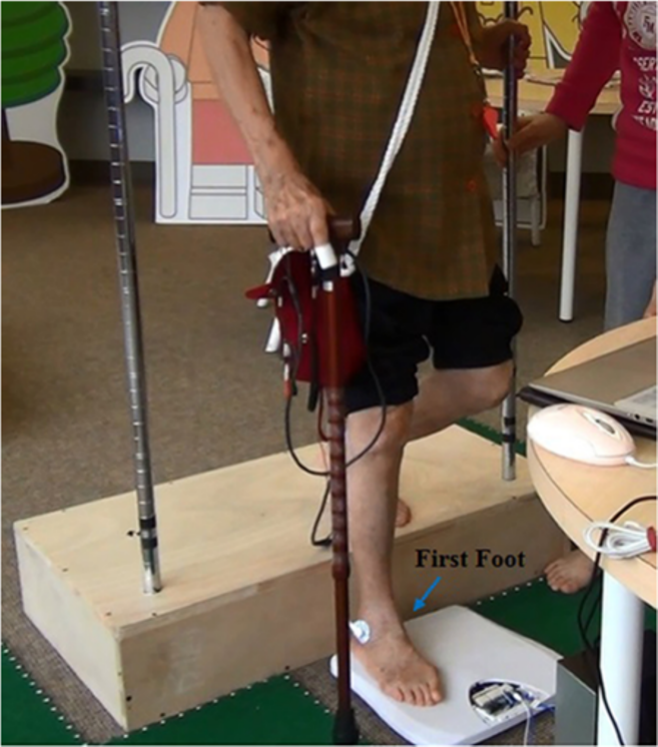
**An illustration of the first foot.**

The EMG signals were transformed in MATLAB to root mean square (RMS) values and then divided by the MAX RMS, which was the maximum activity in the measured part of the muscle, and expressed as RMS(%). The CoP signals were expressed in terms of Stabilometric Parameters, which are described in Table 2. We collected EMG signals from the loading response period, which began when the participant’s feet first contacted the force plate and ended one second later. The first foot contact was defined as when the first foot landed on the force plate (which foot was first depended on the participant’s preference; Figure 2) and the second foot contact was defined as when the second foot contacted the force plate. The CoP signal was recorded from when the first foot touched the force plate and ended when the person was standing squarely with both feet on the force plate. The RMS(%) values are shown as means ± standard deviation. Statistical analyses were performed using Minitab, version 16. Two-way ANOVA was used to examine whether step height or cane length affected muscle load and balance in the study participants. Differences were considered significant if *P* <0.05.

**Table 2:** **Description of stabilometric parameters**

Stabilometric parameters	Description
MDIST	The mean of the resultant distance time series and represents the average distance from the mean center of pressure (CoP)
MDIST_AP	The mean absolute value of the antero-posterior (AP) time series and represents the average AP distance from the mean CoP
MDIST_ML	The mean absolute value of the medio-lateral (ML) time series and represents the average ML distance from the mean CoP
RDIST	The root mean square value of the resultant distance time series
RDIST_AP	The standard deviation of the AP time series
RDIST_ML	The standard deviation of the ML time series
TOTEX	The total length of the CoP path and is approximated by the sum of the distances between consecutive points in the CoP path
TOTEX_AP	The total length of the CoP path in AP direction and is approximated by the sum of the distances between consecutive points in the time series
TOTEX_ML	The total length of the CoP path in the ML direction and is approximated by the sum of the distances between consecutive points in the ML time series
MVELO	The average velocity of the CoP
MVELO_AP	The average velocity of the CoP in the AP direction
MVELO_ML	The average velocity of the CoP in the ML direction
95%CC AREA	The area of the circle with a radios equal to the one-side 95% confidence limit of the resultant distance time series

## Results

### Descriptive statistics

The mean cane lengths used by the study participants were 84.00 ± 5.12 cm (Cane 1), 78.78 ± 5.19 cm (Cane 2), and 74.17 ± 2.75 cm (Cane 3); these results are shown in Table 1.

## EMG Results

**Figure 3:**
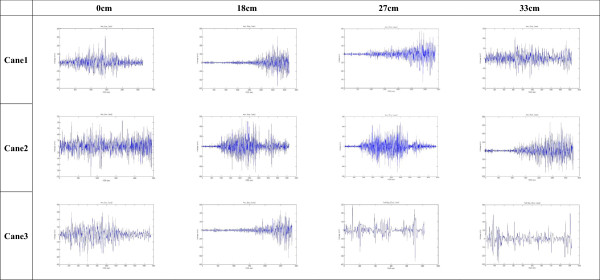
**Arm electromyography signal during each experimental scenario.** x-axis represents time/1,024 (sec) and y-axis represents voltage (mV), which indicates muscle force. One participant was taken as an example.

**Figure 4:**
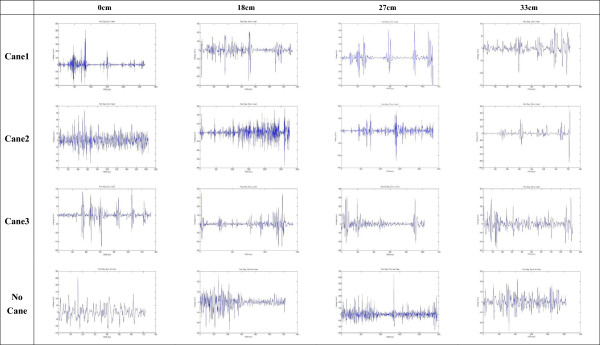
**Leg electromyography signal at the first foot contact during each different experimental scenario.** x-axis represents time/1,024 (sec) and y-axis represents voltage (mV), which indicates muscle force. One participant was taken as an example.

**Figure 5:**
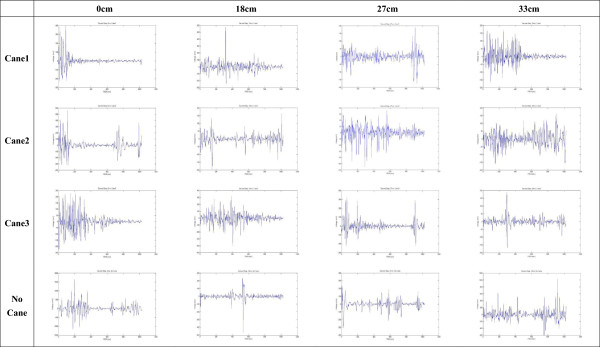
**Leg electromyography signal at the second foot contact during each different experimental scenario.** x-axis represents time/1,024 (sec) and y-axis represents voltage (mV), which can be transferred to muscle force.

**Figure 6:**
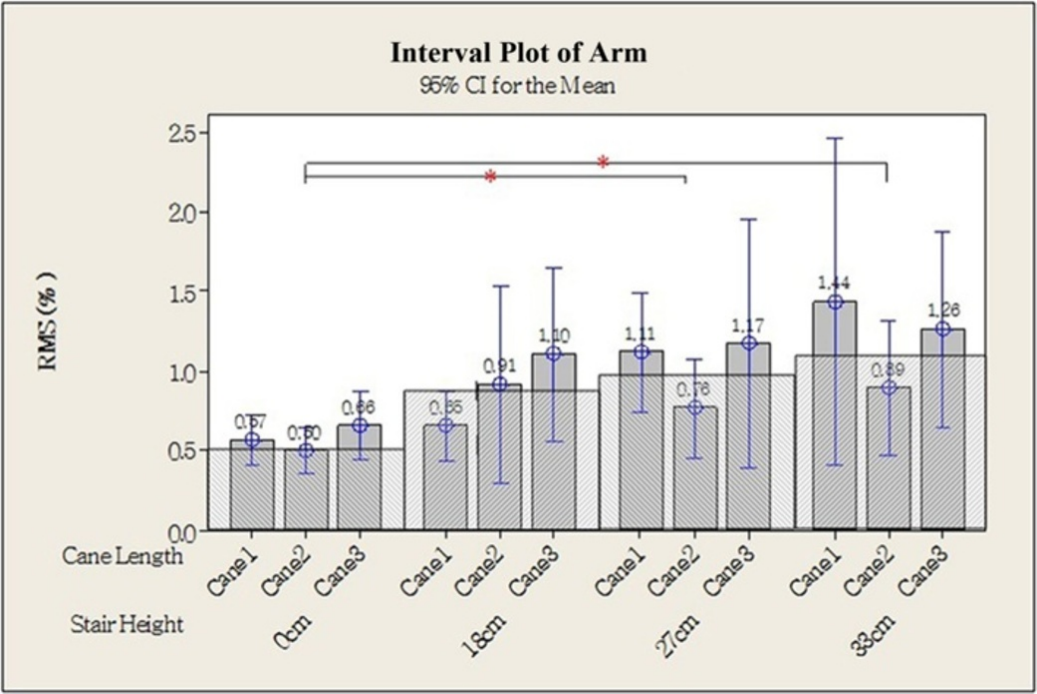
**Root mean square of the arm.** *P <0.05, two-tailed.

**Figure 7:**
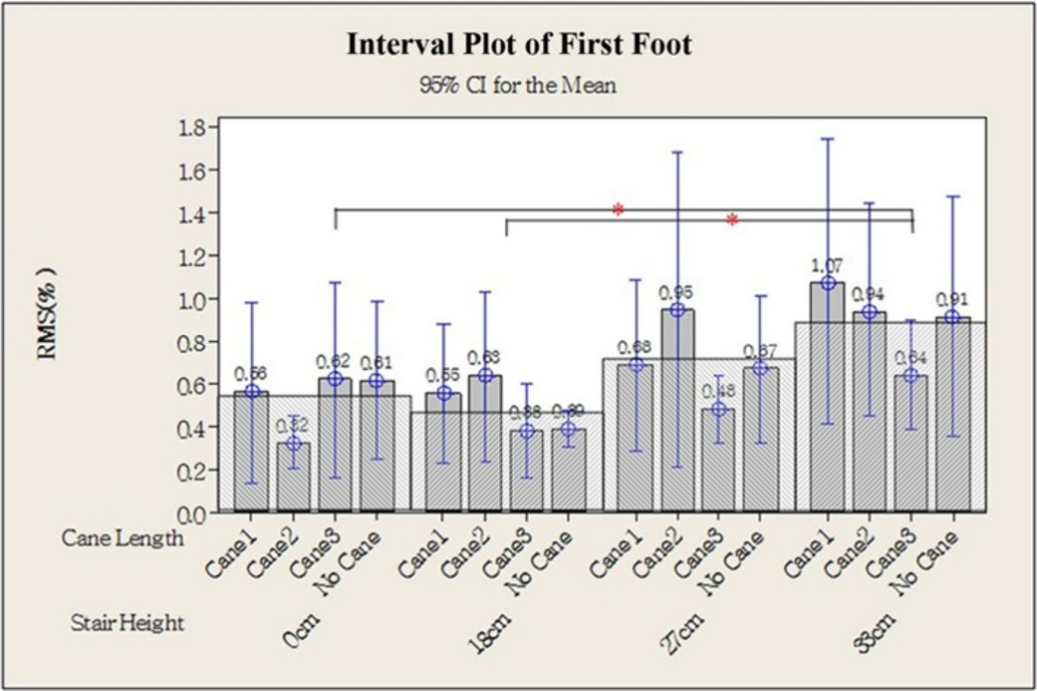
**Root mean square of the gastrocnemius muscle at the first foot contact.** *P <0.05, two-tailed.

Representative EMG signals from one older individual showing the load on the muscles of the arm, on the leg at the first foot contact, and on the leg at the second foot contact are shown in Figures 3, 4, and 5. Statistical analysis of data from all participants revealed that higher steps were associated with greater load on the arm (Table 3), and there were significant differences in RMS between the 0 cm (0.57 ± 0.34) and both the 27 cm (1.01 ± 0.89) and the 33 cm steps (1.17 ± 1.18; Figure 6; *P* <0.05). EMG data from the gastrocnemius muscle at the first foot contact revealed similar results, with the load on the gastrocnemius muscle when stepping down from 33 cm (0.89 ± 0.90) being significantly greater than when stepping down from both 0 cm (0.53 ± 0.70) and 18 cm (0.48 ± 0.51; Figure 7; *P* <0.05); however, the load after stepping down 0 cm tended to be greater than after stepping down 18 cm, although this difference was non-significant. The RMS value for the second step was not significantly affected by step height. Cane length did not significantly affect any RMS values.

**Table 3:** **Root mean square (RMS)(%) of the electromyography signal**

	Arm (RMS)	First foot (RMS)	Second foot (RMS)
**Step height (cm)**			
**0**	0.57* ± 0.34	0.53* ± 0.70	0.63 ± 0.80
**18**	0.89 ± 0.83	0.48 ± 0.51	0.45 ± 0.58
**27**	1.01* ± 0.89	0.70* ± 0.88	0.41 ± 0.34
**33**	1.17* ± 1.18	0.89* ± 0.89	0.46 ± 0.57
**Cane length**			
**Cane 1**	0.92 ± 0.95	0.70 ± 0.87	0.47 ± 0.44
**Cane 2**	0.75 ± 0.68	0.71 ± 0.93	0.49 ± 0.67
**Cane 3**	1.02 ± 0.94	0.52 ± 0.55	0.51 ± 0.72
**No cane**	–	0.64 ± 0.69	0.50 ± 0.57

**Figure 8:**
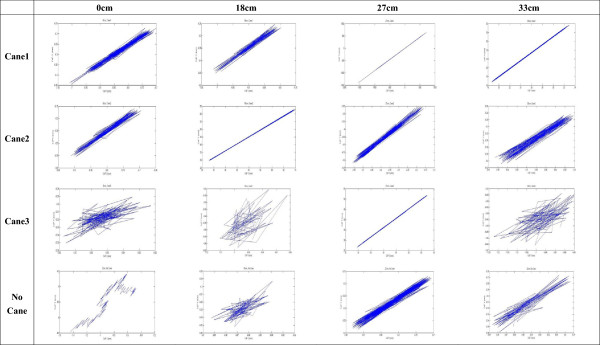
**Center of pressure (CoP) signal during each experimental scenario.** x-axis represents the ML direction (mm) and y-axis represents the AP direction (mm). One participant was taken as an example.

**Figure 9:**
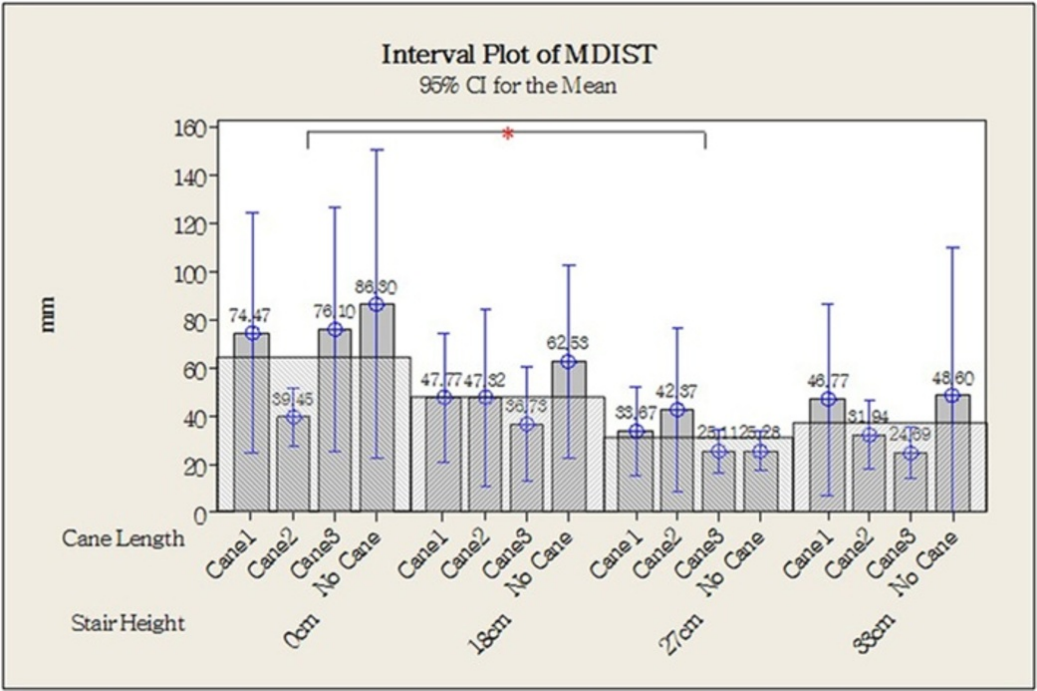
**Center of pressure (COP) value of mean distance (MDIST; with step height as the main factor).** *P <0.05, two-tailed.

**Figure 10:**
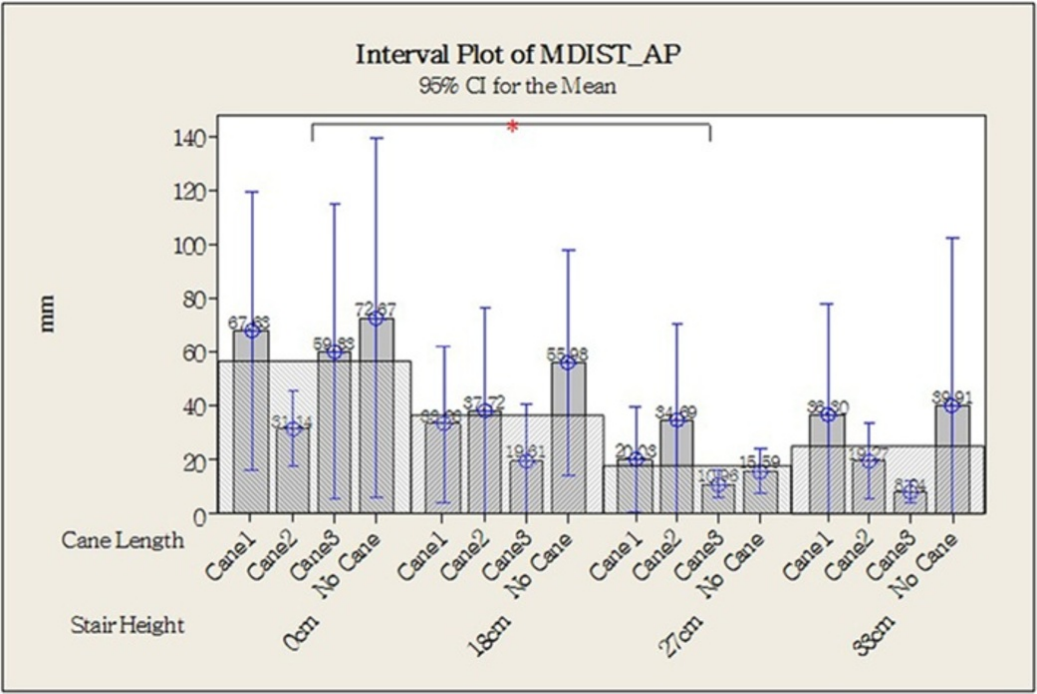
**Center of pressure (COP) value of antero-posterior mean distance (MDIST_AP; with stair height as the main factor).** *P <0.05, two-tailed.

**Figure 11:**
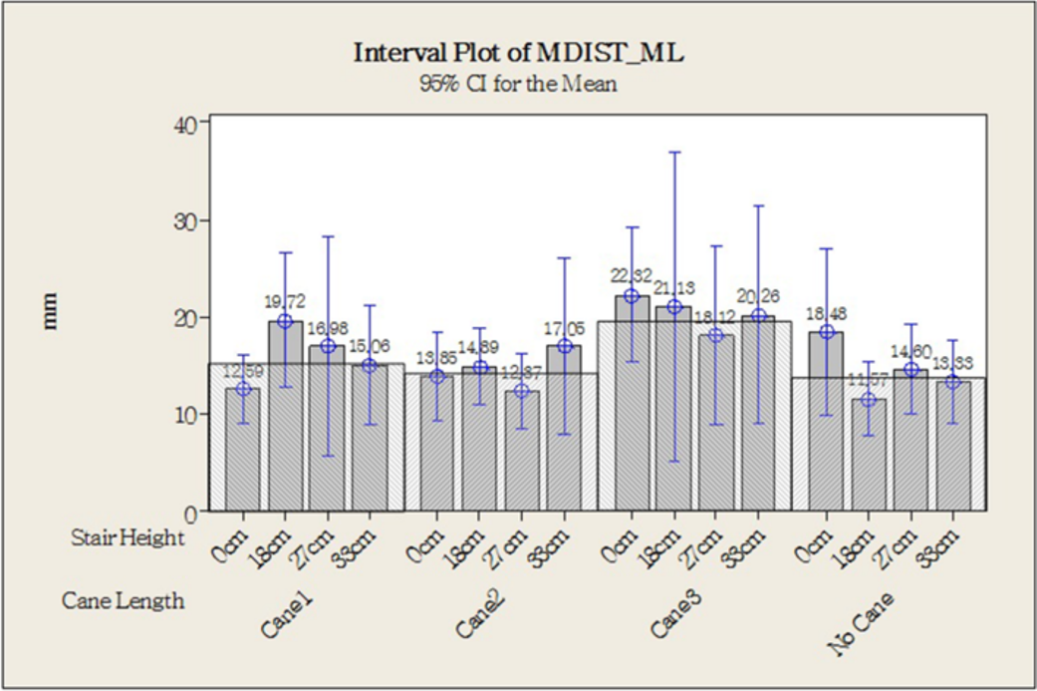
**Center of pressure (COP) value of medio-lateral mean distance (MDIST_ML; with cane length as the main factor).** *P <0.05, two-tailed.

Figure 8 shows the original CoP data plotted for one older individual in each of the different experimental combinations (different step heights and cane lengths). Statistical analysis revealed that the CoP Stabilometric Parameters MDIST, MDIST_AP, TOTEX, TOTEX_AP, MVELO, and MVELO_AP were significantly affected by step height (Table 4). Figures 9 and 10 show the results for the MDIST and MDIST_AP parameters, which indicate that participants swayed more when stepping down 0 cm than when stepping down 27 cm (*P* <0.05); the results were similar for the other parameters. CoP values were not significantly affected by cane length; however, we did observe that Cane 3 resulted in slightly more sway than the other cane lengths as measured by MDIST_ML (*P* = 0.076; Figure 11).

**Table 4:** **Stabilometric parameter center of pressure values**

		MDIST (mm)	MDIST_AP (mm)	MDIST_ML (mm)	RDIST (mm)	RDIST_AP (mm)	RDIST_ML (mm)	
	Stair height (cm)							
	0	69.86*	58.73*	16.67	157.77	148.38	19.24	
	18	49.21	37.3	16.75	118.2	106.78	19.34	
	27	31.21*	19.96*	15.52	56.7	46.71	17.92	
	33	38.1	25.99	16.42	81.29	70.01	18.96	
	Cane length							
	Cane 1	52.18	40.89	16.07	115.68	105.59	18.55	
	Cane 2	40.33	30.85	14.45	89.48	81.63	16.69	
	Cane 3	39.92	23.96	20.35	78.71	62.92	23.49	
	No cane	55.99	46.37	14.49	130.16	121.91	16.73	
	**TOTEX (mm)**	**TOTEX_AP (mm)**	**TOTEX_ML (mm)**	**MVELO (mm/s)**	**MVELO_AP (mm/s)**	**MVELO_ML (mm/s)**	**AREA_CC (mm/s)**	**AREA_SW (mm** ^**2**^ **/s)**
Stair height (cm)								
0	3,394.67*	3,356.67*	65.68	7,000.23*	6,594.67*	97.79	942,720	33,164.3
18	1,782.83	1,739.2	66.02	3,680.02	3,625.57	97.66	675,821	22,019.5
27	892.97*	853.1*	61.08	1,912.16*	1,855.54*	97.69	161,771	8,700.69
33	1,412.08	1,368.99	64.68	3,017.75	2,958.12	97.88	548,671	10,991.6
Cane length								
Cane 1	1,882.77	1,844.39	63.28	4,706.18	4,655.36	97.59	593,516	18,350.6
Cane 2	1,559.83	1,526.06	56.83	3,117.07	3,064.48	97.73	372,360	14,669.7
Cane 3	1,546.71	1,489.46	80.4	2,467.78	2,408.74	97.99	363,491	12,425.7
No cane	2,492.99	2,451.8	56.97	5,284.63	5,231.35	97.71	971,282	29,395.9

*P <0.05, two-tailed.

## Discussion

The EMG signal is a recording of physiological variations in muscle fiber membranes. As muscle contraction increases, the EMG signal amplitude increases [23]. CoP is the location of the vertical reaction vector when a subject stands on a plane [24]. The CoP reflects the orientation of the body, as well as its movement to maintain its center of gravity. A higher CoP indicates that the body might be unbalanced.

### Stair environment

#### EMG

Step height is an important factor that affects both the hip and knee flexors [25]. Müller et al. [26] measured the force on adult joints when ascending or descending three different stair inclination angles, and found that the force on joints increased as the stair inclination angle increased. The difference in force between the minimum and maximum inclinations tested was 14.8%. The results of the current study were similar; the RMS(%) value for the arm showed that participants experienced greater muscle loads when stepping down from a greater height. The same was true of the load on the gastrocnemius muscle at the first foot contact, except for the tendency for less muscle load after stepping down from an 18 cm high step than during level walking (0 cm).

Individuals took multiple steps as a pre-planned strategy in various situations [27], often causing the steps taken after the first step to appear unstable [28]. This study showed that the first step was the key to maintaining balance. Thus, the first step might have been more significantly affected by step height than the second step, as the first step may have a more important role in maintaining stability. Based on the results of the present study and past research, older individuals who selected suitable strategies maintained better balance in different environments.

The ability of a cane to assist with maintaining stability can be observed through the relationship between the EMG signals from the gastrocnemius and the biceps brachii at the time of the first foot contact. Participants used greater arm force to reduce the muscle load on their leg. Joyce and Kirby have previously reported that the use of a cane can reduce pressure on the lower limbs [15]. If older people properly apply force to a cane, it may reduce the force on their feet and legs. This movement strategy may be suitable for older individuals with leg weakness.

#### CoP

The CoP results showed that the sway amplitude during level walking (0 cm) was larger than when descending a 27 cm high step, especially in the AP direction. This corresponds with past research, which showed that the ground reaction forces were smaller when ascending and descending stairs than during level walking, and that step height had a significantly greater effect in the AP than in the ML direction [26]. Lu et al. [29] studied 10 older stroke patients who performed level walking without a cane, and found that sway was more evident in the AP direction than in the ML direction. Thus, it can be inferred that AP sway controls body stability more than ML sway.

### Relationship between EMG and CoP

For the synoptic composites in this study, the EMG and CoP results were similar to those reported previously. First, as stair height increased, muscle load increased. Second, the body can gain greater balance with the application of greater force. In particular, the participants exerted greater force to improve balance as step height increased, maintaining relatively better balance than when stepping down from lower step heights. Observation of the participants during level walking and 18 cm step height conditions revealed that when the participants used slightly more arm force they were able to reduce the muscular load on both legs, as well as to achieve a state of equilibrium superior to that achieved during level walking. Thus, conscious application of arm muscle force could reduce loads on the limb muscles and improve balance. In addition, the participants paid more attention during the different step situations than during level walking, and as a result their balance improved. In short, we suspect that setting suitable obstacles into their daily environment might make older people pay more attention to walking and, therefore, improve their stability. However, a comparison of 27 and 33 cm step heights showed that sway increased as step height increased, despite increases in the forces generated by the arm and both legs. This suggests that descending stairs that are too steep is laborious for older individuals.

### Cane length

#### EMG

Our results indicate that cane length does not affect the force exerted by the arm and both legs when descending a step. When the participants held a cane with their elbow flexed at 20° to 30°, the cane helped offset their body weight. Although there were three different cane lengths, all were within the standard recommendations for cane length, which might explain why cane length had no significant effect in this study. In a previous study, researchers measured heart rate in osteoarthritis patients during walking with different cane lengths and found that there were no significant differences if the cane length was within the suggested measurements [20]. Additionally, the provision of a handrail offered extra assistance, allowing subjects to exert more force than usual on the arm not holding the cane.

#### CoP

Cane length did not have a large effect on balance, having a small effect only on ML parameters such as MDST_ML (*P =* 0.076), RDST_ML (*P =* 0.076), and TOTEX_ML (*P =* 0.076). However, Lu et al. [29] found that stroke patients swayed less when using Cane 2 than when using Cane 1. The selection of subjects, or the variation between the older participants in that study and this one, may have caused this difference in results.

Cane length in our study tended to affect the participants’ stability in the ML direction. Our results showed that participants swayed more when using Cane 3 than the other cane lengths. It can be inferred that using a shorter cane may offer similar stability to longer canes in the AP direction, but does not provide enough ML stability.

As previously mentioned, factors such as the installation of handrails, standard cane lengths, and other variables might have affected the participants’ equilibrium. These factors are as follows: i) Participants did not use their original canes: the canes used in this experiment were supplied by the researchers. They may have felt different from the participants’ usual canes, and therefore caused changes in movement while stepping down. ii) Exercise habits: the participants differed in their normal exercise habits. Therefore, their ability to exert force differed, which may have caused them to use different strategies to walk down stairs. iii) Physiological condition: any unknown disease could have affected the participants’ performance during the experiment; however, individual differences between subjects cannot be avoided. iv) Adaptability: humans are able to adapt well to changes in their environment. Participants might have been affected by the variables in the beginning of the experiment, but they may have gradually acclimated to them and become less affected by step height or cane length.

## Conclusions

The results of this study demonstrate the physiological effects of different cane lengths and step heights on older people. The main results of the study indicated that cane length did not greatly affect the load on the arm or legs when descending a step. If the cane length is within the normally recommended limits, it helps the user gain stability during movement. Further, step height has a greater effect on stability than cane length. Thus, greater step height causes older people to adopt different strategies to improve their stability. When stepping off the 0 and 18 cm steps, increased load on the arm and lower muscle load on the legs resulted in greater stability. However, when stepping down from 27 and 33 cm, body sway was still greater than seen with the lower steps, even though muscle load was also greater.

We hope that this study will provide useful information for other researchers who are also interested in the effects of the stair environment on balance and mobility in older people.

## Abbreviations

AP: Antero-posterior
CoP: Center of pressure
EMG: Electromyography
MDIST: Mean distance
MDIST_AP: Antero-posterior mean distance
MDIST_ML: Medio-lateral mean distance
ML: Medio-lateral
MVELO: Mean velocity
MVELO_AP: Antero-posterior mean velocity
RMS: Root mean square
TOTEX: Total excursions
TOTEX_AP: Antero-posterior total excursions.

## References

[1] Lee CF, Kuo CC (2001). A Pilot Study of Ergonomic Design for Elderly Taiwanese People.

[2] Okada A (1997). Ergonomics approach in universal design. Special Issue JSSD.

[3] Lexell J, Taylor CC, Sjostrom M (1988). What is the cause of the ageing atrophy? Total number, size, and proportion of different fiber types studied in whole vastus lateralis muscle from 15 to 83-year-old men. J Neurol Sci.

[4] Young A, Stokes M, Crowe M (1985). The size and strength of the quadriceps muscles of old and young men. Clin Physiol.

[5] Waters DL, Baumgartner RN, Garry PJ (2000). Sarcopenia: current perspectives. J Nutr Health Aging.

[6] Nevitt MC, Cummings SR, Kidd S, Black D (1989). Risk factors for recurrent nonsyncopal falls. A prospective study. J Am Med Assoc.

[7] Robbins AS, Rubenstein LZ, Josephson KR, Schulman BL, Osterweil D, Fine G (1989). Predictors of falls among elderly people: results of two population based studies. Arch Intern Med.

[8] Sieri T, Beretta G (2004). Fall risk assessment in very old males and females living in nursing homes. Disabil Rehabil.

[9] Daley MJ, Spinks WL (2000). Exercise, mobility and aging. Sports Med.

[10] Colling J, Park D (1983). Home, safe home. J Gerontol Nurs.

[11] McVey LJ, Studenski SA (1988). Falls in elderly. Adv Clin Rehabil.

[12] Blake AJ, Morgan K, Bendall MJ, Dallosso H, Ebrahim SB, Arie TH, Fentem PH, Bassey EJ (1988). Falls by elderly people at home: prevalence and associated factors. J Aging Phys Act.

[13] Graafmans WC, Ooms ME, Hofstee HMA, Bezemer PD, Bouter LM, Lips P (1996). Falls in the elderly: a prospective study of risk factors and risk profiles. Am J Epidemiol.

[14] Tinetti ME, Speechley M, Ginter SF (1988). Risk factors for falls among elderly persons living in the community. N Eng J Med.

[15] Joyce BM, Kirby RL (1991). Canes, crutches and walkers. Am Fam Physician.

[16] Mully GP (1988). Everyday aids and appliances: walking sticks. Br Med J.

[17] Jebsen RH (1967). Use and abuse of ambulation aids. J Am Med Assoc.

[18] Sainsbury R, Mulley GP (1982). Walking sticks used by the elderly. Br Med J.

[19] Dean E, Ross M (1993). Relationships among cane fitting function and falls. Phys Ther.

[20] Jones A, Monteiro Alves AC, de Oliveira LM, Saad M, Natour J (2008). Energy expenditure during cane-assisted gait in patients with knee osteoarthritis. Clinics.

[21] Kim K, Cha YJ (2011). Cane length influence on plantar pressure distribution of adult Hemiplegia patients. J Phys Ther Sci.

[22] DeLisa JA, Currie DM, Gans BM, Gatens PF, Leonard JA, McPhee MC, Lippincott JB (1993). Rehabilitation Medicine: Principle and Practice.

[23] Komi PV (1992). Stretch-shortening cycle. Strength and Power in Sport.

[24] Winter DA (1991). Biomechanics and Motor Control of Human Gait: Normal, Elderly and Pathological.

[25] Müller R, Bisig A, Kramers I, Stüssi E (1998). Influence of stair inclination on muscle activity in normals. J Biomech.

[26] Riener R, Rabuffetti M, Frigo C (2002). Stair ascent and descent at different inclinations. Gait Posture.

[27] Luchies CW, Alexander NB, Schultz AB, Ashton-Miller J (1994). Stepping responses of young and old adults to postural disturbances: kinematics. J Am Geriatr Soc.

[28] Mcllroy WE, Maki BE (1996). Age-related changes in compensatory stepping in response to unpredictable perturbations. J Gerontol.

[29] Lu CL, Yu B, Basford JR, Johnson ME, An KN (1997). Influences of cane length on the stability of stroke patients. J Rehabil Res Dev.

